# Venous Thromboembolism While on Anticoagulation With Apixaban

**DOI:** 10.7759/cureus.15189

**Published:** 2021-05-23

**Authors:** Farah Mazahreh, Fuad Habash, Angel López-Candales

**Affiliations:** 1 Cardiology, University of Arkansas for Medical Sciences, Little Rock, USA; 2 Cardiovascular Medicine, University of Missouri Kansas City, Kansas City, USA

**Keywords:** venous thromboembolism (vte), saddle pulmonary embolism, failed anticoagulation, cardiovascular diseases / blood

## Abstract

Venous thromboembolism (VTE) is a common condition whose pathophysiology is explained by Virchow’s triad with stasis, hypercoagulability, and endothelial injury. Direct oral anticoagulants (DOACs) showed non-inferiority when compared with conventional treatment using subcutaneous low molecular weight heparin (LMWH) and warfarin, but treatment failure is a concern and remains a challenge for physicians. In our case report, we present a patient who had VTE in the form of a saddle pulmonary embolus while on apixaban.

## Introduction

Venous thromboembolic disease occurs when thrombus develops within the deep veins, followed by propagation and dislodgment of the thrombus causing a pulmonary embolism, which occurs in one-third of patients with deep vein thrombosis (DVT) [1]. The high mortality rate of pulmonary embolism (PE) even among young adults [2] and the possibility of developing acute right ventricular failure makes clinical suspicion, early diagnosis, and management of paramount importance. Current guidelines for the prevention of PE in those with a previous history of DVT expanded to include treatment with direct oral anticoagulants (DOACs) such as apixaban (Anti-factor Xa) [3]. Although major trials like Apixaban for the Initial Management of Pulmonary Embolism and Deep-Vein Thrombosis as First-Line Therapy (AMPLIFY) [4] and RECOVER™ II [5] for apixaban and dabigatran, respectively, confirmed the efficacy of these medications with significant advantages over conventional treatment, it is still to be known if cases with treatment failure warrant further investigation of the safety and efficacy of the newer medications.

## Case presentation

A 73-year-old male patient with diabetes, hypertension, hyperlipidemia, and a history of unprovoked deep venous thrombosis on apixaban 5 mg twice daily, with no evidence of DVT resolution at that time, presented to the hospital with shortness of breath for three days. The initial episode of shortness of breath was associated with chest pain and diaphoresis that lasted for 30 minutes. The patient was compliant with his medication and active at home. He had no family history of recurrent thrombosis.

On physical examination, the patient was in mild respiratory distress. Vital signs showed a heart rate of 95 bpm, respiratory rate of 20/minute, and oxygen saturation of 87% on room air. Cardiac examination showed normal first (S1) and second (S2) heart sounds, no added abnormal cardiac sounds, and, in particular, there was no appreciable splitting of S2. The respiratory examination was unremarkable.

**Figure 1:**
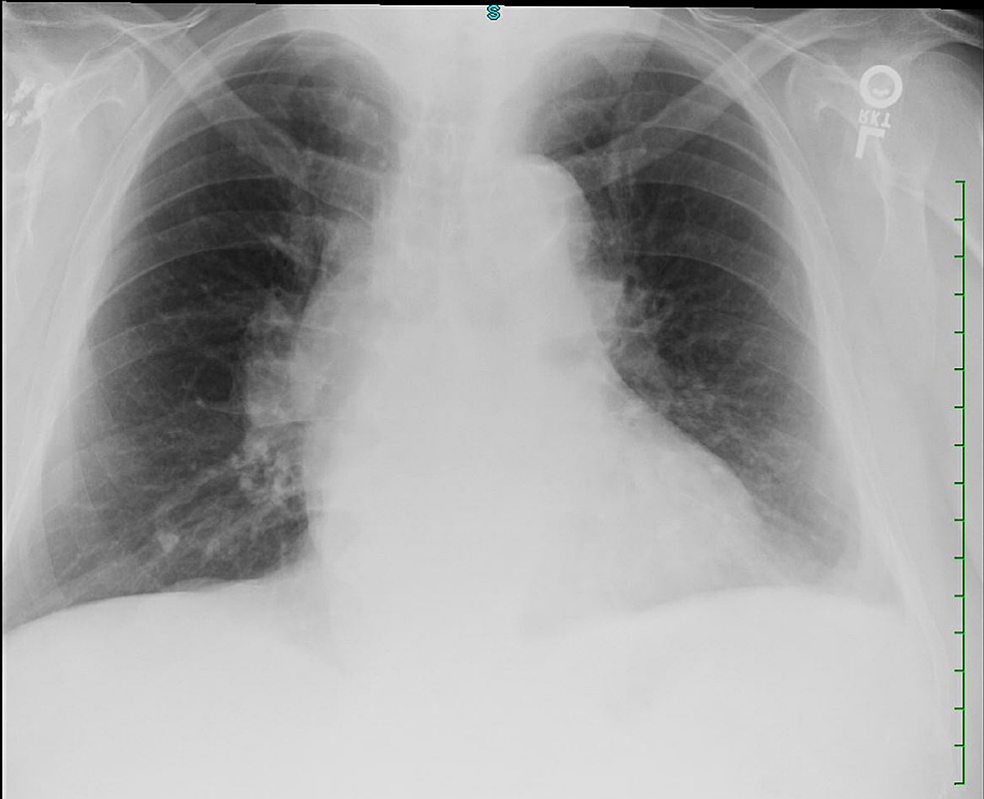
Chest X-ray showing borderline cardiac silhouette with a small left pulmonary effusion but without pulmonary infiltrates or consolidation

Brain natriuretic peptide (BNP) was elevated (358.6 pg/ml; Ref: </=100 pg/mL), initial troponin I was elevated (0.114 ng/ml; Ref 0.0-.028 ng/ml), and lactic acid was elevated (3.8 mmol/L; Ref 0.5-2 mmol/L). International normalized ratio (INR), activated partial thromboplastin time (aPTT), platelet count, renal function, and electrolytes were within the normal range. Electrocardiography (ECG) was significant for T-wave inversions in anterior leads (V1-3), with a rightward shift of the QRS axis but otherwise no ST-segment deviation. Coronavirus disease 2019 (COVID-19) testing using nucleic acid amplification test (NAAT) for severe acute respiratory syndrome coronavirus 2 (SARS-CoV-2) was negative. Chest X-ray (Figure 1) showed increased cardiac silhouette, small left pleural effusion, and no evidence of pulmonary consolidation.

**Figure 2:**
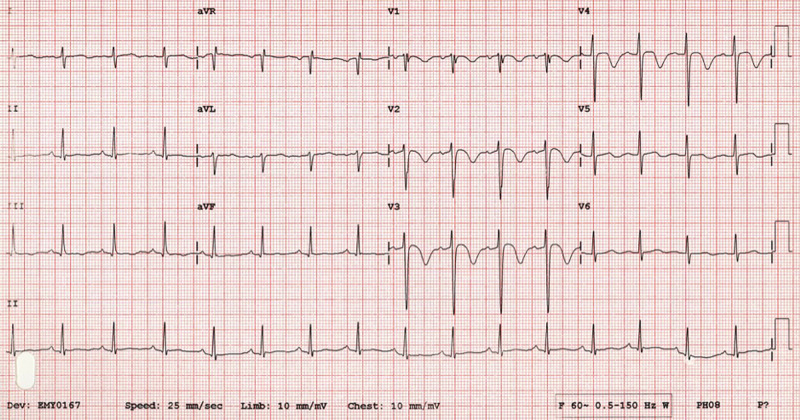
Electrocardiogram: type B Wellens

Myocardial ischemia was on the differential diagnosis list, with ECG findings concerning for acute coronary syndrome (ACS), including T-wave inversion and ST-segment depression, which may well be PE masquerading as myocardial infarction (MI). Considering the patient’s presentation, ECG findings (Figure 2) of Wellens type B (deeply inverted T-waves in V2-V3, which is 89% specific for significant stenosis of the left anterior descending artery) [6] and a previous history of coronary calcification on a previous lung CT scan, ACS had to be excluded and cardiology was consulted for a possible percutaneous coronary angiogram (PCI).

**Figure 3:**
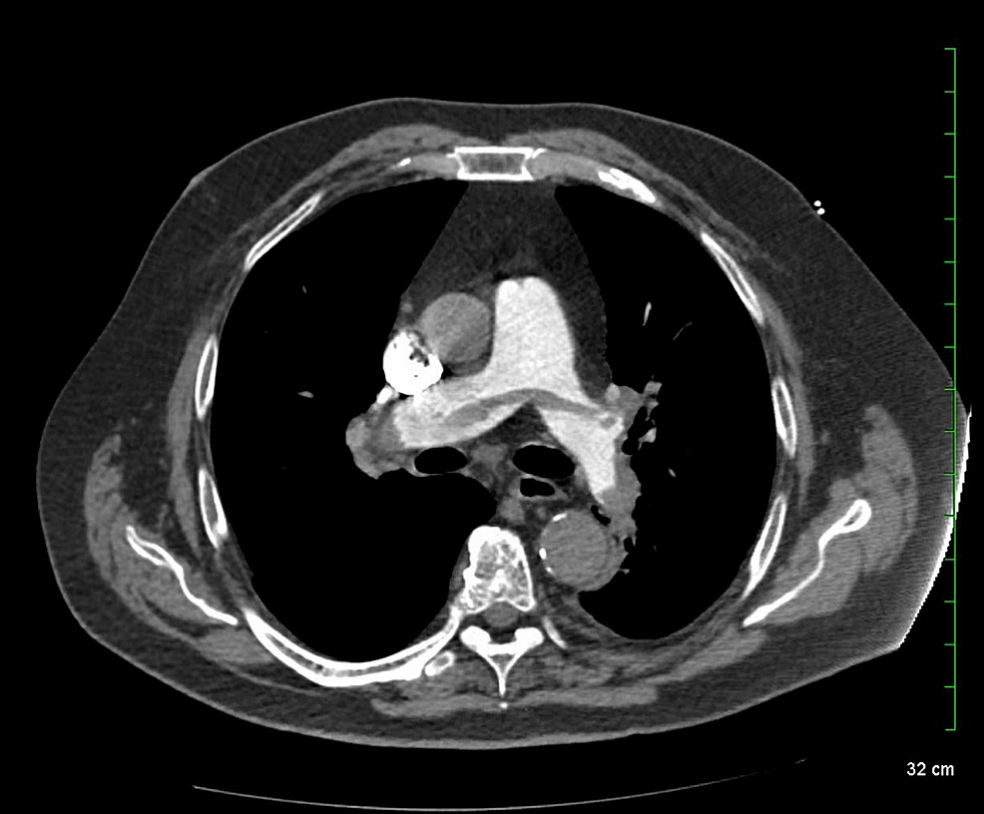
CT-pulmonary embolus protocol: saddle embolus with bilateral occlusive and non-occlusive emboli in both the right and left branches of the pulmonary artery

In the meantime, an initial bedside echocardiogram was technically difficult due to the patient’s body habitus but revealed right ventricle dilation with right ventricular strain pattern and mild global systolic dysfunction (ejection fraction of 45%-50%). With the current echocardiographic findings, pulmonary embolus was high on our differential. The CT-pulmonary embolus protocol with contrast showed a large saddle embolus with bilateral occlusive and non-occlusive emboli in both the right and left pulmonary arteries (Figure 3). A formal echocardiogram was negative for both regional wall abnormalities and patent foramen oval with agitated saline.

Patient’s management included supplemental oxygen, initiation of un-fractionated heparin infusion as per protocol, and admission to the intensive care unit. CT chest, abdomen, and pelvis showed no evidence of malignancy, and hyper-coagulability testing was negative as well.

## Discussion

Pulmonary embolism leads to a partial or complete blockage of pulmonary artery branches, most commonly due to embolization of a leg deep venous thrombus [1]. Pulmonary embolus is the third most common cause of cardiovascular death [7].

PE can be classified into massive (hemodynamic instability), sub-massive (right ventricular dysfunction on echocardiogram), and non-massive (without hemodynamic instability or right ventricular dysfunction). Diagnosis can be made by CT-PE showing filling defects in the pulmonary artery and its branches. Management depends on classification; it was shown that systemic thrombolytic therapy improves survival in patients with massive PE. It can also be used in sub-massive PE but there is a considerable risk of bleeding. In addition, it was proven that the mortality rate in those treated with heparin anticoagulation is less than 3%, which makes it the treatment of choice in sub-massive PE [8].

Ekosonic Endovascular System (EKOS) was then introduced, potentially reducing the risk of bleeding. It is catheter-based pharmacochemical thrombolysis that employs high-frequency ultrasound to augment the penetration of thrombolytics into selective targets [9]. It is indicated in patients with contraindication to fibrinolysis or when risk stratification in hemodynamically stable patients suggests increased morbidity and mortality [10].

Treatment of VTE depends on the long-term risk of recurrence after discontinuing anticoagulation; the duration varies between provoked VTE versus unprovoked VTE. Patients with provoked PE or with transient risk factors, such as major surgeries, immobilization, prolonged air travel, direct trauma to the leg, or hormone contraception, can be treated for three months based on the fact that the recurrence risk is 1% in the first year and 0.5% annually thereafter. On the other hand, patients with persistent risk factors or unprovoked PE should be treated indefinitely, with regular assessment of bleeding risk and anticoagulation benefit [11].

Recently, the use of warfarin has significantly reduced with increasing physician and patient preference for DOACs. Apixaban is an oral factor Xa inhibitor; it is a drug with rapid onset of action and predictable pharmacokinetics that allow a fixed-dose regimen. With these distinguishing properties, apixaban became a convenient and alternative to conventional therapy with vitamin K analog (warfarin) and subcutaneous low molecular weight heparin (enoxaparin). This was enforced by the AMPLIFY study [4], which showed noninferiority to warfarin and significantly decreased bleeding risk in the treatment of acute deep vein thrombosis.

Our patient had saddle pulmonary embolus while on the appropriate dose of anticoagulation with apixaban, which constitutes treatment failure. A decision was made to treat the patient with heparin infusion due to the urgent need for tight monitoring of drug levels with readily available laboratory assays (activated partial thromboplastin time) to ensure the therapeutic goal. Interventional radiology was consulted but the patient did not qualify for thrombolytic treatment.

Treatment failure of thromboembolism was previously mentioned in the literature, with an incidence rate of 2% [12-13]. One case report published in 2016, of a patient treated with different classes of DOACs, failed to resolve pulmonary clots who needed final treatment with warfarin [14]. Another case was reported in 2017 with recurrence of DVT while on apixaban. Although the absence of clinically available assays to evaluate medication plasma level and activity is not always associated with treatment failure, it was proposed by researchers [15]. Several attempts were made to correlate the effect of DOACs on coagulation laboratory studies, but the results are variable and need more understanding for validation [16].

In the Aristotle trial [17], apixaban plasma levels were measured using the liquid chromatography/mass spectrometry (HPLC/MS-MS) method, as well as the chromogenic anti-Xa method. Laboratory measurements detecting anti-Xa activity were considered useful for the quantification of drug levels for apixaban and rivaroxaban. It was shown that undetected anti-Xa activity excludes significant drug levels.

In comparison with other DOACs, the acute DVT [18] study, part of the EINSTEIN program, showed that recurrent venous thromboembolism (fatal pulmonary embolism, nonfatal pulmonary embolism, and recurrent deep venous thrombosis) all occurred while following patients being treated with rivaroxaban as well. Further management varies but considering a medication that can be tightly monitored for adequate drug levels is a valid alternative [15]. According to a single-center review [13] of 59 patients who failed treatment with DOACs, it was a common practice to switch to therapeutic low molecular weight heparin (LMWH) followed by another DOAC after successful parenteral therapy.

Increasing the dose of DOAC is another approach; in the RENOVATE clinical trial [12], a comparison between dabigatran etexilate 220 mg and dabigatran etexilate 150 mg showed that the composite of total VTE (symptomatic and venographic), in addition to the mortality rate, was 6% and 8.5%, respectively, suggesting a dose-dependent decrease in the incidence of VTE while receiving dabigatran. On the other hand, patients who developed VTE while receiving rivaroxaban did not benefit from dose escalation [19]. This might indicate that increasing the dose is more effective before developing treatment failure.

A metanalysis of reported cases of treatment failure on DOACs observed that patients who had recurrent VTE had an underlying antiphospholipid syndrome, atrial fibrillation, or DVT [20]. Thus, the mechanism of failure may be in part due to an underlying condition, insufficient dose, or the different pharmacokinetics and pharmacodynamics of each DOAC [20].

It is certain that the risk of VTE recurrence is decreased with apixaban when compared with placebo, but for a subset of the population being treated with Apixaban, the recurrence of VTE or VTE-related death was 2.3% and the mortality rate was 1.5%. The reported causes of death were PE, PE not ruled out, cancer, cardiovascular disease, and infectious causes [4]. Young adults have high mortality as well, and it has been increasing in comparison to the past decade [2]. Therefore, it is of paramount importance to diagnose recurrent VTE because any delay in detection and treatment would significantly increase mortality. 

## Conclusions

The emerging cases of recurrent VTE raise suspicion for the efficacy of DOACs. This challenges the concept of the patients' safety while on anticoagulants, promoting awareness about the condition. Such treatment failure should always be on the differential diagnosis of patients presenting with chest pain and shortness of breath even if they are appropriately anti-coagulated. In the future, perhaps, a clinically available test for the activity of DOACs would help decrease the number of similar cases.
